# An Italian Multicenter Study on the Epidemiology of Respiratory Syncytial Virus During SARS-CoV-2 Pandemic in Hospitalized Children

**DOI:** 10.3389/fped.2022.930281

**Published:** 2022-07-14

**Authors:** Raffaella Nenna, Luigi Matera, Amelia Licari, Sara Manti, Gaia Di Bella, Alessandra Pierangeli, Anna Teresa Palamara, Luana Nosetti, Salvatore Leonardi, Gian Luigi Marseglia, Fabio Midulla, ICHRIS Group, Massimo Agosti

**Affiliations:** ^1^Department of Maternal Infantile and Urological Sciences, Sapienza University of Rome, Rome, Italy; ^2^Pediatric Clinic, Fondazione IRCCS Policlinico San Matteo, Pavia, Italy; ^3^Department of Clinical, Surgical, Diagnostic and Pediatric Sciences, University of Pavia, Pavia, Italy; ^4^Pediatric Respiratory and Cystic Fibrosis Unit, Department of Clinical and Experimental Medicine, San Marco Hospital, University of Catania, Catania, Italy; ^5^Department of Pediatrics, Pediatric Sleep Disorders Center, F. Del Ponte Hospital, Insubria University, Varese, Italy; ^6^Laboratory of Virology, Department of Molecular Medicine, Affiliated to Istituto Pasteur Italia, Sapienza University of Rome, Rome, Italy; ^7^Laboratory Affiliated to Istituto Pasteur Italia-Fondazione Cenci Bolognetti, Department of Public Health and Infectious Diseases, Sapienza University of Rome, Rome, Italy; ^8^Department of Infectious Diseases, Istituto Superiore di Sanità, Rome, Italy

**Keywords:** respiratory infections, pediatrics, respiratory syncytial virus, SARS-CoV-2, COVID-19

## Abstract

Since the beginning of 2020, a remarkably low incidence of respiratory virus hospitalizations has been reported worldwide. We prospectively evaluated 587 children, aged <12 years, admitted for respiratory tract infections from 1 September 2021 to 15 March 2022 in four Italian pediatric hospitals to assess the burden of respiratory viruses during the COVID-19 pandemic in Italy. At admission, a Clinical Respiratory Score was assigned and nasopharyngeal or nasal washing samples were collected and tested for respiratory viruses. Total admissions increased from the second half of October 2021 to the first half of December 2021 with a peak in early November 2021. The respiratory syncytial virus (RSV) incidence curve coincided with the total hospitalizations curve, occurred earlier than in the pre-pandemic years, and showed an opposite trend with respect to the incidence rate of SARS-CoV-2. Our results demonstrated an early peak in pediatric hospitalizations for RSV. SARS-CoV-2 may exhibit a competitive pressure on other respiratory viruses, most notably RSV.

## Introduction

Respiratory viruses, and among them Respiratory Syncytial Virus (RSV), cause a large burden of respiratory diseases, accounting for most pediatric emergency visits and hospitalization worldwide, with high healthcare costs and significant morbidity (1). In the pre-Coronavirus Disease (COVID-19) era, RSV used to have a significant impact on children < 5 years, causing about 3.2 million hospital admissions globally (2) with annual winter epidemics peaking between December and February in the Northern Hemisphere (3). This trend is confirmed by other studies, including our previous published work (4, 5). Remarkably low incidence of respiratory viruses hospitalizations, with flat epidemiology, has been reported worldwide since early 2020, during the COVID-19 pandemic (5–8). Reduction in communicable disease was the lucky another side of the coin of coronavirus preventive measures, such as face masks use, hand washing, social distancing, and a ban on the mass gatherings (9).

A matter of concern was a possible resurgence of respiratory viruses diseases since spring-summer 2021, likely driven by relaxed community lockdown coupled with waning population immunity with a consequent increase in population susceptibility (8, 10, 11). Recently, data from the Southern Hemisphere reported a temporal shift in 2021 RSV seasonality that was not balanced by a more severe clinical presentation (10, 12). Despite the unusual onset of the RSV epidemic in the United States during summer, the relative timing of the RSV epidemic between states followed the usual spatial pattern (from East to North and West) (13). In Italy, normal epidemics showed a similar pattern (from North to South), following the changes in weather conditions, with peak RSV activity correlating with cold temperatures and higher relative humidity (14). Demonstrating the impact of SARS-CoV-2 periodic waves and the effects of COVID-19 preventive measures on the epidemiology of other respiratory viruses and anticipating epidemic timing for RSV can make policy decisions aimed at containing the morbidity and the spread of these viruses and at planning RSV passive and, eventually, active prophylaxis more effective.

For this study, we combined data sources from 4 major pediatric Hospitals, representative of the North, Center, and South of Italy, to examine trends in respiratory viruses hospitalization in autumn-winter 2021–2022 in Italy. We aimed at registering the resurgence of respiratory viruses diseases and describing the spatial variation in epidemic timing in Italy, during the COVID-19 pandemic. Moreover, we superimposed the incidence of COVID-19-related admissions, extracted from the national update of the Italian Superior Institute of Health to the incidence of RSV-related admissions to evaluate the relationship between viruses.

## Materials and Methods

We prospectively evaluated 587 children, aged < 12 years, consecutively admitted for respiratory tract infections from September 1, 2021, to March 15, 2022, at four Italian Pediatric University Hospitals: (1) The University of Insubria in Varese (North); (2) University of Pavia (North); (3) Sapienza University of Rome (Center) and (4) University of Catania (South). The Italian peninsula is divided into three different regions depending on the different latitudes, from continental Europe to the borders of Africa: the North, with a colder continental climate; the Center, with a more temperate climate; the South, with a warmer climate. This is reflected in the spread of viruses, which are particularly affected by climatic conditions. Thus, including in this study four centers from different Italian regions (Varese and Pavia for the North, Rome for the Center, and Catania for the South), we were able to evaluate not only the general trend of the RSV epidemic but also its spread throughout the Italian peninsula. The total number of hospitalized children due to respiratory tract infections was enrolled in the participating hospitals, even those with comorbidities.

This prospective observational study was approved by the Ethics Committees of the recruiting centers. Informed consent was waived, as the analysis was performed on de-identified data. Demographic and clinical data were collected from patients’ clinical charts. A Clinical Respiratory Score (CRS) was assigned at admission. The score included the child’s color, respiratory rate, presence of wheeze, use of accessory muscles, mental status, and oxygen saturation, and each variable ranges from 0 to 2. Thus, CRS total score ranges from 0 to 12 and defines into three categories: Mild (<3), Moderate (4–7), and Severe (8–12) (15).

From all children, we collected a nasopharyngeal (NP) washing (NPW) (in infants up to 1 year of age) or from NP swabs (NPS) (for older children). The children underwent NPW or NPS within 24 h of hospitalization. To test RSV and other respiratory viruses, the four centers used different PCR-based molecular methods, either commercially available kits or homemade real-time PCRs. In the latter tests, a sample was considered positive to a viral target when its Ct value was < 40. In particular, in Varese, a qualitative multiplex real-time RT- PCR intended for testing RSV, hRV, and human influenza type A and B (FluA and FluB), was performed. In Pavia, detection of RSV RNA was performed with a specific one-step real-time RT-PCR assay targeting RSV-A and -B together; hRV, FluA and FluB, human metapneumovirus (hMPV), human parainfluenza virus (hPiV) type 1–4 and human adenoviruses (hAdV) were tested using a panel of laboratory-developed real-time RT-PCR, previously validated and tested (16). In Rome, purified RNA from respiratory samples was retrotranscribed using random-primers; cDNA was tested by real-time PCRs (17) to detect and subtype RSV and by home-made, qualitative PCRs for FluA and FluB, human coronavirus h (CoV) OC43, 229E, NL-63 and HUK1, hAdV, hRV, hPiV type 1–3, human bocavirus (hBoV) and hMPV (18). In Catania, the rapid molecular test for testing RSV, FluA, and FluB (Xpert^®^ Xpress, Cepheid) or the R-GENE^®^ qualitative multiplex real-time RT- PCR (Biomerieux) were used.

Data on the weekly incidence of COVID-19-related admissions per 1,000,000 inhabitants, were extracted from the national update of the Italian Superior Institute of Health, published on March 16, 2022 (19).

We analyzed the data using SPSS version 27 (IBM Corp., New York, United States). Continuous variables were described as means ± standard deviations and categorical variables as frequencies and percentages. Comparisons between continuous variables were assessed using the analysis of the Variance test and categorical variables using the Chi-Square test. A *p*-value < 0.05 was considered statistically significant.

## Results

We consecutively enrolled 587 children admitted for respiratory tract infection from the North to the South of Italy: 105 (17.7%) in Varese, 130 (22.1%) in Pavia, 218 (37.1%) in Rome, and 134 (22.8%) in Catania. Children had a median age of 0.6 years (IQ:0.18–2.2) and 309/587 (52.6%) were males.

Concerning the CRS, the mild forms of respiratory diseases were predominant (54%), while in 39.7% of patients they were moderate, and in 6.3% they were severe. Table 1 shows the main characteristics of the population studied according to the participant centers (Table 1).

**TABLE 1:** The main characteristics of the population studied in the participant centers.

	Center				Total
		
	Varese	Pavia	Rome	Catania	
N. of case	105	130	218	134	587
Age < 5 years, *n* (%)	105 (100)	124 (96.1)	211 (96.8)	108 (81.2)	548 (93.7)
Male sex, *n* (%)	48 (45.7)	71 (54.6)	131 (60.1)	59 (44.0)	309 (52.6)
Family history for asthma, n (%)^a^	26 (24.8)	18 (15.8)	33 (20.0)	35 (26.3)	112 (21.7)
Virus^b^					
RSV (+), *n* (%)	76 (72.4)	54 (49.1)	114 (54.3)	43 (32.1)	287 (51.3)
hRV (+), *n* (%)	1 (1.0)	19 (17.3)	16 (7.6)	6 (4.5)	42 (7.5)
Other viruses (+), *n* (%)	2 (1.9)	20 (18.2)	3 (1.4)	16 (11.9)	41 (7.3)
Severe cases, *n* (%)^c^	14 (13.3)	9 (6.9)	5 (2.3)	9 (6.7)	37 (6.3)
Epidemic peak (week number)	42–43	46–47	46–47	48–49	46–47
Latitudine	45°49′N	45°12′N	41°54′N	37°30′N	

*^a^Parental asthma.*

*^b^Viruses are: RSV, Respiratory Syncytial Virus; hRV, human Rhinovirus.*

*Other viruses = Adenoviruses, Bocavirus, Metapneumovirus.*

*^c^Severe cases are defined as having CRS = 8.*

**FIGURE 1:**
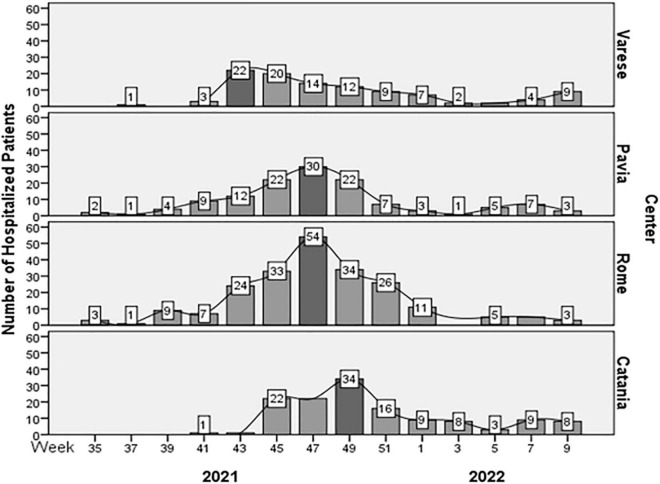
Distribution of hospitalized patients for respiratory diseases from September 1, 2021 to March 15, 2022. The epidemic peaks are highlighted in dark gray.

Dividing our observational period into weeks’ timeframes, the total admissions for respiratory diseases increased from the second half of October 2021 to the first half of December 2021 with a peak at the beginning of November 2021 (Figure 1).

Considering viral etiology, RSV (*n* = 306, 52.1%) was the most frequent identified virus in the four participant centers (Varese: *n* = 76, 72.4%; Pavia: *n* = 65, 50%; Roma: *n* = 122, 55.9%; Catania: *n* = 43, 32%). RSV incidence curve coincided with the total hospitalizations curve.

When we analyzed the single centers’ data, we found that the peak in admissions occurred earlier in the North of Italy, in Varese, and, subsequently, it spread southwards, through Pavia, Rome, and Catania, according to latitude and different climatic conditions (*p* < 0.01) (Table 1 and Figure 1). In all centers, the RSV epidemic peak occurred earlier than in pre-pandemic years, during which the earliest RSV-associated hospitalizations occurred in mid-December (13).

**FIGURE 2:**
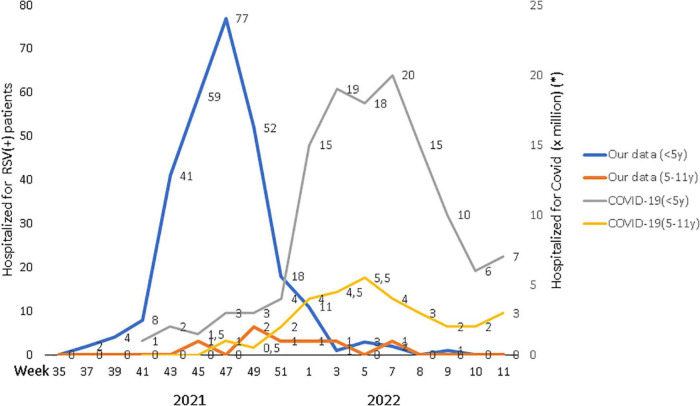
Pediatric admissions for RSV + respiratory diseases by age vs. COVID-19 + admissions during the epidemic timeframe 2021–2022. COVID-19 admissions were collected from the national update of ISS (18).

When we compared RSV admissions incidence to the weekly hospitalization rate for COVID-19, we found that they had an opposite trend (*p* < 0.001). RSV circulation had a surge in early autumn and peaked in November 2021, while the low activity of SARS-CoV-2 was registered in children; on the contrary, RSV incidence was drastically reduced when the novel Omicron (B.1.1.529) variant of SARS-CoV-2 started to circulate in children (Figure 2).

Finally, considering CRS, we found that the mild forms were prevalent: mild 317 (54%), moderate 233 (39.7%), and severe 37 (6.3%).

## Discussion

In this study, we reported an unseasonal RSV circulation that lasted from early autumn to early winter 2021 and was apparently blunted by the arrival of the novel SARS-CoV-2 variant. That delay did not affect the timing pattern of respiratory viruses spreading from North to South in Italy. To the best of our knowledge, we are the first to report an abrupt drop in this early RSV epidemic season and to hypothesize a competition between RSV and SARS CoV-2 in children.

Several factors may have contributed to the unseasonal RSV epidemiology, as well as that of other respiratory viruses. An explanation could come from restrictive measures adopted to slow the SARS-CoV-2 pandemic that dramatically reduced childhood respiratory infections, particularly RSV, except for rhinovirus (5). As expected, a resurgence of respiratory viruses was registered, when lockdown measures were relaxed. Moreover, a possible decline in the population immunity due to the disappearance of respiratory viruses during winter 2020–2021 with a possible increase in population susceptibility may have contributed to the rapid increase in cases in early autumn. Therefore, the re-emergence of respiratory viruses was not-unexpected during the COVID-19 pandemic. However, the abrupt decrease of cases by late December and the ending of RSV season by early January, concurrently with a sudden increase of SARS-CoV-2 pediatric cases, was unexpected (20). To understand this phenomenon, we examined the epidemiological curve of SARS-CoV-2 cases in Italy and noted that the surge of pandemic cases in fall was delayed with respect to other European countries with the exception of Spain, probably due to climatic conditions. Our data shows that the sharp decrease in RSV cases paralleled the SARS-CoV-2 surge during December 2021. One of the possible explanations is that a viral interference phenomenon may explain the sudden RSV disappearance observed in Italy. It has been long hypothesized and then proved in different experimental contexts (21, 22) that a respiratory viral infection could prevent the super-infection of other respiratory pathogens due to the activation of the innate immunity that confers to respiratory mucosal cells the ability to counteract a second virus replication, mainly through the interferon response (23). Other data supporting viral interference come from epidemiological studies; it has been well documented that the circulation of Influenza Virus H1N12009 during the first pandemic winter has been delayed by the Rhinoviruses (hRVs) cases in September-October in several countries and other large studies followed (23, 24). Similarly, during the 2009 influenza pandemic, an average delay of 0.58 months in the onset of RSV season was reported (25). Moreover, hRVs circulation in autumn can also influence RSV epidemic seasons (26, 27). Recently, it has been demonstrated that hRV triggers an interferon response that blocks SARS-CoV-2 replication (28). Influenza Virus, but not RSV, reduced SARS-CoV-2 replication and in turn, SARS-CoV-2 interfered with RSV-A replication in Nasal Epithelial Cells *in vitro* (29). Accordingly, it is possible to hypothesize that RSV circulation this year was halted by the ongoing spread of the SARS-CoV-2 Omicron variant surge that was particularly contagious and abundant in unvaccinated children (30). Further studies are needed to investigate this possibility.

Finally, our results showed that, regarding CRS, the mild forms were predominant. This finding let us speculate that there is no evidence for a possible overlap of the immune response against RSV and SARS-CoV-2 and their mutual enhancement, leading to a worse clinical picture.

## Conclusion

In conclusion, our multicenter study demonstrated an early and intense peak in RSV-associated pediatric admissions. Considering that SARS-CoV-2 is becoming endemic, its circulation will affect that of other respiratory viruses, and vice-versa. Reliably predicting the onset of the RSV epidemic, has become a major challenge for those involved in preventing respiratory infections. Clinicians need to be prepared for the advent of respiratory viruses to timely use healthcare sources and effectively plan RSV passive and, eventually, active prophylaxis.

## Data Availability Statement

The original contributions presented in this study are included in the article/supplementary material, further inquiries can be directed to the corresponding author.

## ICHRIS (Italian Children Respiratory Infections Surveillance) Group

Massimo Agosti, Guido Antonelli, Fausto Baldanti, Flaminia Bonci, Maria Giulia Conti, Greta Di Mattia, Guglielmo Ferrari, Antonella Frassanito, Ginevra Gargiulo, Federica Giardina, Manuela Lo Bianco, Fabrizio Maggi, Paola Magri, Enrica Mancino, Matteo Naso, Federica Novazzi, Giuseppe Oliveto, Giuseppe Fabio Parisi, Maria Papale, Paola Papoff, Laura Petrarca, Antonio Piralla, Santiago Presti, and Gaia Vanzù.

## Author Contributions

RN, LM, and FM: conceptualization, project administration and supervision. RN, AL, SM, GD, LM, AP, ATP, LN, SL, GM, FM, and ICHRIS Group: data curation and writing—review and editing. LM and RN: formal analysis and writing—original draft. All authors contributed to the article and approved the submitted version.

## Conflict of Interest

The authors declare that the research was conducted in the absence of any commercial or financial relationships that could be construed as a potential conflict of interest.

## Publisher’s Note

All claims expressed in this article are solely those of the authors and do not necessarily represent those of their affiliated organizations, or those of the publisher, the editors and the reviewers. Any product that may be evaluated in this article, or claim that may be made by its manufacturer, is not guaranteed or endorsed by the publisher.
